# Society Reports—Medico Chirurgica Society

**Published:** 1878-11-20

**Authors:** R. C. Word


### SOCIETY REPORTS.

### MEDICO CHIRURGICAL SOCIETY.

REPORTED BY DR. R. C. WORD.

Monday evening, Nov. 4th., Dr. Crawford in the Chair.

Dr. Thos. S. Powell reported a case of yellow fever. The patient was a mulatto man, who came from Chattanooga on the 6th of October. On the 7th he felt somewhat unwell. On the 8th he met up with some friends, and indulged freely in intoxicating drink. I was called to see him on the 9th, and found him with considerable fever—pulse 110. He had some frontal headache; the tongue coated brown, with red margins; the eye presenting a yellowish tinge, and there was severe pain in the epigastric region, and in the abdomen.

His recent arrival from Chattanooga led me to suspect yellow fever, and I prescribed accordingly. A hot foot-bath was ordered; applications of mustard was made to the stomach and bowels, and frictions made upon the spine. At the first evidence of a decline of the fever, the sulphate of quinine was administered in full doses, until twenty-four grains were taken. On the evening of the 10th there was some fever with delirium. At 12 o'clock at night, quinine was again given, and repeated until twenty-four grains had been given by 8 o'clock on the morning of the 11th. At this time there was no fever, and the patient seemed in all respects better, t ough he complained of some abdominal pain, which was attributed to a purgative administered by his wife before I saw him. For this symptom morphine was prescribed. On the morning of the 12th he was still free from fever, and complained of nothing, save a feeling of prostration. He was directed to take some soup and boiled milk as nourishment, and to keep comfortably covered in bed.

By reason of a call to the country, I did not see him again until 10 o'clock at night, and found that an unfavorable change had occurred. His mind was wandering, and there was hemmorrhage from the bowels, with hiccough and vomiting, of a glairy substance. He was evidently sinking. Efforts to stimulate and revive him proved unavailing, and he expired before morning.

Dr. P. remarked that this being a case of the same kind that prevailed at Chattanooga, and which was pronounced yellow fever by competent authority, constitutes the third case of the disease seen and treated by him. His experience, therefore, was limited.

The shades of difference presented by this disease in different epidemics, are many and perplexing. Even in the same locality, and sometimes in the same ward the different phases which it presents, lead the physician to believe that several different types of fever are prevailing, ranging from the mildest intermittent to the most malignant forms of billious remittent and congestive fevers; and indeed there would appear to be a most intimate relation, if not actual identity, between the ordinary malarial fevers and yellow fever. Cases presenting all the symptoms of billious remittent fever have been seen to merge into the yellow fever, and it is affirmed that yellow fever has been known to glide into the mildest quotidian intermittent, yielding readily to the sulphate of quinine.

The existence of these facts has led to much difficulty in classifying the disease in question, and in determining the precise character of its cause.

For the reasons mentioned, and the fact that the disease has always prevailed in tropical and miasmatic climes, and is arrested by frost—the destroyer of miasm. I incline to the view of those who place it under the head of remittent fever and hold to the miasmatic origin of yellow fever; the severe and unusual effects of the miasm being due to the modifying influences of heat, moisture and local eminations. The precise nature of the eminations remains yet to be discovered Having these views of the disease, I treated the three cases I saw with quinine, and with success, except in the case just reported, and this case, I believe, would also have recovered, had it not been for the depressing influences of previous dissipation, and the fact that I was not able on account of absence to push the treatment with sufficient vigor on the last day.

*Dr. R. C. Word*—Do I understand you to hold that yellow fever is but an aggravated form of billious remittent fever? If so, how do you account for the contagiousness of the disease? I would like you to state your views also in regard to the altitude theory or to the impossibility of its existence beyond the height of a few hundred feet above the level of the sea.

*Dr. Powell*—I do hold that yellow fever should be classed in our nosology as a form of remittent fever, and I think that post mortem results bear me out in this view. Between yellow fever and remittent fever there is difference only in degree, due, as before stated, to the modifying influences of heat, moisture, and local eminations; the increased virulence of the poison being evinced in the more marked and decided manifestations of the same pathological conditions of engorged liver, enlarged spleen, and disintegrating effects of blood poison, producing prostration, black vomit and death. In regard to the altitude theory, I remark; that while it is true that high altitudes are far less favorable to those local eminations, which, in conjunction with malaria, are essential to the production of the disease, yet it has occurred in rare instances, that yellow fever has existed at very elevated points. On one occasion, in Spain, it is said to have reached an altitude of 2,000 feet above the sea level. In this country, however, I am not aware that it has ever extended above a height of six or seven hundred feet: Though some of the local conditions might exist at those high elevations yet that all of them should occur, is not likely, and when they do, must be for brief periods, and must constitute an exception to a general rule. And we believe that cities thus favorably located, need never have the disease, if due attention be given to sanitary regulations. Indeed it is highly probable that even in New Orleans, and at like points, if rigid and unceasing attention was given to sanitary regulations the disease would never occur. This is indicated in the fact that the disease seldom repeats itself in the same place the year immediately succeeding its first appearance, even where the general conditions of atmosphere appear the same, due, we believe, to the special and rigid attention given to sanitary reform at such times.

Dr. A. R. Alley said he thought the yellow fever at Chattanooga was different from the disease as he had seen it on the coast. He regarded it as an imported disease, and seems in some way influenced by a salt atmosphere. Remittent fever prevailing at the same time, may assume some of its peculiarities. The disease appeared first in Barbadoes in 1647. Its first appearance in this country was at Boston, in 1693—at Charleston in 1699. It has visited Charleston more often than at any point in U. S.

*Dr. R. C. Word.*—I have nothing new to present upon the subject under discussion. While I admit that the views of Dr. Powell are very plausible in regard to the malarious origin of the disease, yet I incline to the view that there is a specific yellow fever germ, requiring for its development, growth and fructification, a continued high temperature; hence is indigenous to tropical or warm latitudes. That it is usually imported to this country in ships, is, I think, beyond question, and yet I doubt not, that at low, warm sea levels on our coast, it may, and has, occasionally originated. The germs having once developed, will propagate and spread in an atmosphere of favorable temperature and conditions. The germs or the atmosphere containing them may be carried in badly ventilated ships, and in railroad cars, or may be absorbed by water, or may be carried in boxes or chests of clothing, and being turned loose among those who are predisposed by a previous warm and unwholesome atmosphere will develop the disease in their midst.

There are some facts in the history of the disease in this country which indicate that the germ or remote cause of the disease may be developed in close, ill-ventilated or confined quarters.

The hold of ships containing filth and bilge water, seems to be its favorite lurking place, and it may be authentically shown that in 1806 the disease originated in the penitentiary, in the suburbs of Richmond, Virginia, at a time when there was no epidemic existing in the adjacent towns, and when the city of Richmond itself was in a healthy condition.

In regard to the change of malarious fevers into yellow fever, and vice versa, it is probable that the different malarial influences prevailing in the same locality may impress their peculiarities on each other, or, blending together, may form mixed or mongrel types of disease.

What an eminent author has styled the "Epidemic Constitution of the Atmosphere" by which the ordinary diseases of the country are impressed with the prevailing epidemic influence, must have been observed by every practitioner of experience, and to my mind explains the facts upon the point mentioned.

